# Genetic parameters of milk β-hydroxybutyrate as an indicator of metabolic diseases in Spanish dairy cows

**DOI:** 10.3168/jdsc.2025-0841

**Published:** 2025-10-25

**Authors:** M.A. Pérez-Cabal, I. Cervantes, J.P. Gutiérrez, N. Charfeddine

**Affiliations:** 1 Animal Production Department–Veterinary Faculty, Complutense University of Madrid, 28040 Madrid, Spain; 2 CONAFE, Confederación de Asociaciones de Frisona Española, 28340 Valdemoro, Spain

## Abstract

Ketosis is the most prevalent metabolic disorder in dairy herds, and reducing its incidence is a key objective in most dairy cattle breeding programs. Whereas clinical ketosis is rarely recorded, milk β-hydroxybutyrate (mBHB) concentration is routinely available through milk recording systems and can serve as a reliable indicator of subclinical ketosis. The objective of this study was to evaluate mBHB as a metabolic disease trait for inclusion in the Spanish Holstein breeding program, aiming to identify animals less susceptible to ketosis by exploring alternative trait definitions for future genetic evaluations. Five mBHB traits were studied, one for each of the first 5 lactations. Two datasets were compared to assess the benefit of using either all available test-day records or only the first test-day available within the initial 68 DIM, which reduced the data volume to 49% of total records. The statistical model included fixed effects of lactation-age at calving, month of calving, and DIM as a linear covariate. Random effects included herd-year of calving, the additive genetic effect, and the permanent environmental effect of repeated measures (test-day records) within lactation. Heritability estimates for mBHB traits ranged from 0.04 to 0.12, with consistent results across both datasets. Genetic correlations among lactations were high (0.67–0.98), 2 test-day records yielded better predictions, mean EBV reliabilities for sires ranged from 12% to 42%, and moderate rank correlations (0.12–0.52) among sires were observed. These findings suggest that using 2 test-day records and data from the first 3 lactations may be sufficient for incorporating mBHB as an indicator trait of subclinical ketosis in the Spanish Holstein breeding program.

## Highlights

• Ketosis is the most prevalent metabolic disease in dairy herds.
• Milk β-hydroxybutyrate showed genetic variability as a subclinical ketosis indicator.
• Genetic selection to reduce ketosis is possible with data from the first 3 lactations.

## Graphical Abstract

**Figure:**
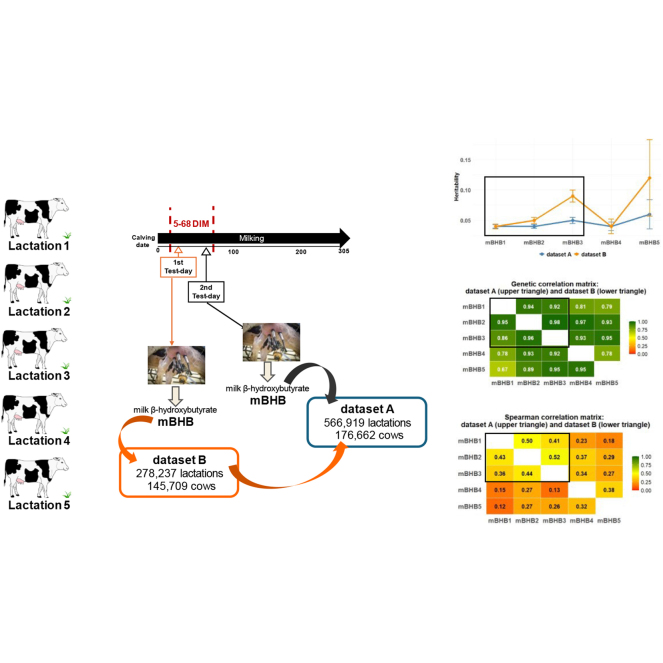
**Summary:** Ketosis is one of the most prevalent metabolic disorders in dairy herds and typically occurs during early lactation due to a negative energy balance, the subsequent mobilization of adipose tissue reserves, and the accumulation of ketone bodies. Milk β-hydroxybutyrate (mBHB) concentration is used as an indicator of subclinical ketosis, as it can be routinely recorded in the milk recording system. This study estimated the genetic parameters of mBHB across 5 lactations, compared sire rankings, and evaluated the minimum test-day records for implementing efficient routine genetic evaluations. The 5 mBHB traits showed low heritability but high genetic correlations. The estimated breeding value reliabilities and rank correlations among sires suggest that using 2 test-day records within the first 68 days in milk from the first 3 lactations is preferable for incorporating mBHB as an indicator trait of subclinical ketosis resistance in the Spanish Holstein breeding program. Cow image by OpenClipart-Vectors from Pixabay; close-up image by Wolfgang Ehrecke from Pixabay.

Disease resistance is a key objective in most dairy cattle breeding programs, with ketosis being one of the primary conditions targeted, as it is the most prevalent metabolic disorder in dairy herds. It arises from the cow's inability to adapt to the energy deficit occurring during the transition from gestation to lactation (Herdt, 2000). After calving, dairy cows inevitably experience weight loss as feed intake fails to meet the high energy demands of milk production. Consequently, cows must mobilize adipose tissue reserves. This state of negative energy balance lasts around 2 mo after calving (Kauppinen, 1983). Although most cows adapt to this period without issue, those that do not may develop hyperketonemia or clinical/subclinical ketosis (Herdt, 2000) due to abnormal concentrations of nonesterified fatty acids and ketone bodies in the blood, which also alter milk composition (Benedet et al., 2019). Beyond the direct effects of the disease on health, fertility, and milk yield, ketosis increases the risk of secondary disorders such as fatty liver, displaced abomasum, milk fever, lameness, and mastitis, all of which contribute to significant economic losses (Rico and Barrientos-Blanco, 2024). For these reasons, metabolic health has become a key component of breeding goals in modern dairy cattle selection programs.

Ketosis can be classified as clinical or subclinical, depending on the presence or absence of visible symptoms. Cows affected by clinical ketosis show severe loss of body condition, a characteristic ketone smell in breath, reduced feed intake, decreased milk yield, and general weakness. In contrast, subclinical ketosis does not present visible symptoms and may therefore remain undetected and untreated, while still negatively affecting productivity (Baird, 1982). Subclinical ketosis can be diagnosed through the measurement of ketone body concentrations, particularly BHB, the most prevalent and stable ketone body in bovine fluids (Nielsen et al., 2003). It can be detected either in blood or milk samples. Whereas blood sampling is invasive and time-consuming, mBHB can be routinely measured in test-day milk samples using Fourier-transform infrared spectroscopy (de Roos et al., 2007; van der Drift et al., 2012a), offering a stress-free and practical alternative. The thresholds used to define hyperketonemia based on mBHB concentrations vary widely, ranging from 0.01 to 0.20 mmol/L (Benedet et al., 2019), depending on the prediction model. The heritability estimates for mBHB are also variable (0.04 to 0.16), as reported by Benedet et al. (2019), and depend on lactation stage and parity. This genetic variation in metabolic adaptation to negative energy balance supports the use of mBHB as an indicator trait in breeding programs selecting for high-producing cows with low susceptibility to subclinical ketosis in dairy cows (van der Drift et al., 2012b; Rius-Vilarrasa et al., 2018).

Because the risk period for subclinical ketosis in early lactation may include 2 test-day records, it would be of interest to evaluate how this information should be statistically handled to assess the potential benefits of using one or both mBHB records per lactation, instead of considering mBHB as different traits by stage of lactation as proposed by Koeck et al. (2014). The aim of this work was to study mBHB as an indicator trait of metabolic diseases to be incorporated into the Spanish Holstein breeding program. Specifically, the study focused on 2 main goals: (1) to estimate the genetic parameters of mBHB and (2) to define the best-performing mBHB traits that minimize computational requirements for routine genetic evaluations.

All information regarding mBHB was obtained from the official milk recording system and provided by the Spanish Holstein Association (CONAFE). Most Spanish autonomous milk recording organizations in Spain offer screening for mBHB in test-day milk samples.

Cows included in the study had their first calving between 2013 and 2023, and only data from lactations 1 to 5 were considered. Calving ages ranged from 18 to 40 mo in lactation 1, from 28 to 59 mo in lactation 2, from 38 to 77 mo in lactation 3, from 48 to 94 mo in lactation 4, and from 58 to 110 mo in lactation 5. Test-day observations of mBHB recorded between 5 and 68 DIM were considered, as this interval corresponds to the first official test-day after calving and coincides with the period of highest risk of hyperketonemia. The editing of mBHB phenotypes as the response variable analyzed was as follows: a constant was added to all mBHB concentrations to avoid negative and zero values, setting the minimum record at 0.001 mmol/L, before performing a log_10_-transformation to achieve normality. Five traits were used as response variables: mBHB in each of the first 5 lactations. After data editing, the database had to be reduced from the total initial number of records because of the high computational demands of the statistical analyses. Following common practice in dairy genetic evaluations when herd-year is included as a fixed effect in the model, a minimum of 20 records per herd-year group was required for each trait, which is considerably higher than the threshold of ≥5 records per herd-year group suggested by Vasconcelos et al. (2008). Data editing was carried out using R software (version 4.2.1, R Core Team, 2022).

Two datasets were compared for the study of minimal computational requirements: Dataset A included all mBHB test-day records per lactation within the 5 to 68 DIM period (56% of lactations had information on 2 test-days), comprising 566,919 lactations from 176,662 cows across 1,696 herds. Dataset B included only the first available test-day record per lactation and represented 49% of the lactations contained in dataset A. A 10-generation pedigree was constructed using the R package *pedigreeTools* (Vazquez et al., 2023), comprising 509,226 animals. Univariate and bivariate analyses were performed using ASReml-R 4.2 (Butler et al., 2023). The animal linear mixed model included the fixed effects of DIM as a covariate, month of calving (12 levels), and lactation-age (up to 44 levels). Random effects included were herd-year of calving (to avoid unstable or poorly informed estimates arising from very small contemporary groups for lactations 4 and 5), the additive genetic effect of (509,226 animals), and a random effect to consider up to 2 test-day records per lactation.

The criteria used to compare results from dataset A and dataset B were the mean squared error (**MSE**), to evaluate the prediction of the 5 trait observations from the univariate or bivariate analyses, and the EBV reliability and ranking correlations for sires of cows within datasets. The MSE was calculated as the average squared difference between observed and predicted values. The EBV reliability was calculated from the standard error of EBV and the additive genetic variance of mBHB estimated by the corresponding analyses. Sires with at least 10 daughters in the corresponding dataset were considered for comparison of sire ranking for mBHB traits across different lactations (2,878 sires in dataset A and 2,085 sires in dataset B). Sire ranking comparison was assessed by estimating Spearman's rank correlation coefficients between EBV from univariate and bivariate analyses. Standard errors of the correlations were estimated using a bootstrap approach with 1,000 replicates (Efron and Tibshirani, 1986).

Regarding genetic parameters, our results suggest strong genetic continuity beyond the third parity. Estimated heritabilities of mBHB traits ranged from 0.04 to 0.12. Estimates obtained from dataset A and dataset B (Table 1) were generally consistent, except for mBHB5, where differences were likely due to the 75% reduction of observations in lactation 5 of dataset B with respect to those in dataset A, and therefore, results for BHB5 should be taken carefully. Heritability in our study tended to slightly increase with parity number. Preliminary analyses in Spanish dairy cows reported similar or slightly lower heritabilities, ranging from 0.04 in the first lactation to 0.03 in third or later lactations (Cervantes et al., 2023), contrary to our findings, which may be attributed to the different modeling approaches and our more restrictive data editing process. Similarly to those results, Klein et al. (2020) reported heritabilities of 0.04 for both first and second lactations, and 0.03 for the third lactation, based on mBHB concentrations from the first test-day (5–42 DIM). Most of the studies in literature report moderate heritability estimates for mBHB, ranging from 0.10 to 0.16. For instance, Lee et al. (2016) estimated heritabilities of 0.10 for the first and second lactations, but only 0.04 for the third lactation within the first 30 DIM. In contrast, Koeck et al. (2014) estimated higher heritability estimates across different lactation stages (first three 20-d intervals), reporting values of 0.14, 0.15, and 0.18, respectively, within the same lactation. Genetic correlations between adjacent lactations were, in general, as expected. The closer the parity number, the higher the genetic correlation. In this regard, correlations estimated between the first and second lactations were high, above 0.94, and between the second and third lactations were higher (0.96–0.98). For the third lactation, the genetic correlations with later lactations were consistently above 0.92. Jamrozik et al. (2016) reported a lower correlation of 0.35 between the first and later lactations, whereas CRV Coop (2020) reported correlations of 0.82 between the first and the second lactations, 0.86 between the first and third, and 0.94 between the second and subsequent lactations, in agreement with our results. In primiparous cows, metabolic processes are still adapting to the dual demands of growth and milk production, which increases environmental variance and reduces the proportion of phenotypic variance explained by additive genetics. In contrast, multiparous cows face a greater metabolic challenge due to higher milk yields and more pronounced negative energy balance, which amplifies the expression of genetic differences in metabolic efficiency (Pryce et al., 2016). The genetic correlations of mBHB across lactations are high because the genetic potential adaptation to the negative energy balance is determined by the same underlying metabolic processes. The expression of genes involved on metabolic efficiency, hormonal regulation, and the liver's capacity to metabolize fatty acids remains relatively stable across lactations, which explains the consistent genetic predisposition to ketosis (Soares et al., 2021).

**Table 1:** Number of records (n_A_, n_B_), heritabilities, genetic correlations of milk β-hydroxybutyrate (mBHB) concentration traits by lactation (indicated by numbers in the trait abbreviation), and mean squared error (under genetic parameters), based on dataset A (upper diagonal and upper triangle) and dataset B (lower diagonal and lower triangle)^1^

Trait	mBHB1, n_A_ = 234,370	mBHB2, n_A_ = 183,673	mBHB3, n_A_ = 101,479	mBHB4, n_A_ = 39,651	mBHB5, n_A_ = 7,746
mBHB1, n_B_ = 128,330	0.04 (0.003)	0.94 (0.017)	0.92 (0.021)	0.81 (0.052)	0.79 (0.109)
	0.110	0.004	0.004	0.005	0.007
	0.04 (0.004)				
	0.106				
mBHB2, n_B_ = 89,711	0.95 (0.020)	0.04 (0.003)	0.98 (0.012)	0.97 (0.023)	0.93 (0.094)
	0.006	0.004	0.004	0.005	0.007
		0.05 (0.005)			
		0.006			
mBHB3, n_B_ = 44,183	0.86 (0.035)	0.96 (0.020)	0.05 (0.005)	0.93 (0.034)	0.95 (0.067)
	0.006	0.006	0.098	0.05	0.096
			0.09 (0.010)		
			0.092		
mBHB4, n_B_ = 14,064	0.78 (0.096)	0.93 (0.065)	0.92 (0.056)	0.04 (0.007)	0.78 (0.141)
	0.006	0.006	0.006	0.100	0.007
				0.04 (0.012)	
				0.098	
mBHB5, n_B_ = 1,949	0.67 (0.162)	0.89 (0.126)	0.95 (0.098)	0.95 (0.167)	0.06 (0.024)
	0.009	0.009	0.094	0.009	0.077
					0.12 (0.063)
					0.077

1 Standard errors of genetic parameters are shown in parentheses.

Calculated MSE indicated that predictions were slightly better for dataset A when using bivariate analyses, whereas univariate analyses performed better when based only on the first test-day observation (Table 1). This pattern is likely explained by the higher genetic correlations estimated when using all available information within the 5 to 68 DIM period. Mean EBV reliabilities of sires (Table 2) were very similar regardless of the dataset, and bivariate analyses generally yielded slightly higher reliabilities compared with single-trait analyses. Maximum reliability from the univariate analyses was achieved for first lactation (43%–44%), whereas it increased to 52% by performing bivariate analyses between first and second lactations in both datasets. The lowest reliabilities were observed for mBHB4 and mBHB5, due to the few data available for fourth and fifth lactations in both datasets, reflected in the higher standard errors for all estimates, and consequently, in the reliabilities. Spearman correlations between EBV estimated from single-trait and bivariate analyses are shown in Table 3. The highest correlations were observed between consecutive lactations from first to third, ranging from 0.41 to 0.52 in dataset A and 0.36 to 0.44 in dataset B. Dataset A yielded slightly higher correlations, due to the greater amount of data available, although overall trends were consistent across both datasets. Focusing on the first 3 lactations, ranking of sires was better performed using mBHB information from 2 test-days per lactation.

**Table 2:** Overall mean (±SE) reliability (%) of sire EBV for milk β-hydroxybutyrate (mBHB) concentration traits across lactations (indicated by the numbers in the trait abbreviation), estimated from single-trait analyses (diagonal) and bivariate analyses (off-diagonal), based on dataset A (upper diagonal and upper triangle) and dataset B (lower diagonal and lower triangle)

Item	mBHB1	mBHB2	mBHB3	mBHB4	mBHB5
mBHB1	44 ± 0.33	52 ± 0.34	51 ± 0.34	46 ± 0.33	44 ± 0.33
	43 ± 0.36				
mBHB2	52 ± 0.37	41 ± 0.32	51 ± 0.34	45 ± 0.33	42 ± 0.32
		41 ± 0.35			
mBHB3	49 ± 0.37	50 ± 0.38	38 ± 0.31	41 ± 0.33	40 ± 0.32
			39 ± 0.35		
mBHB4	44 ± 0.36	45 ± 0.37	41 ± 0.37	25 ± 0.24	30 ± 0.26
				19 ± 0.22	
mBHB5	43 ± 0.36	42 ± 0.36	40 ± 0.36	24 ± 0.26	15 ± 0.17
					12 ± 0.17

**Table 3:** Spearman rank correlations of sire EBV for milk β-hydroxybutyrate (mBHB) concentration traits across lactations (indicated by the numbers in the trait abbreviation), based on dataset A (upper triangle) and dataset B (lower triangle)^1^

Item	mBHB1	mBHB2	mBHB3	mBHB4	mBHB5
mBHB1	—	0.50	0.41	0.23	0.18
mBHB2	0.43	—	0.52	0.37	0.29
mBHB3	0.36	0.44	—	0.34	0.27
mBHB4	0.15	0.27	0.13	—	0.38
mBHB5	0.12	0.27	0.26	0.32	—

1 Standard errors were lower than 0.0008.

All analyses were also conducted with herd-year fitted as a fixed effect to mimic official genetic evaluations (results not shown). Estimates of genetic parameters, MSE, and Spearman correlations were similar, whereas EBV reliabilities were slightly reduced using the fixed effect (by ∼2 percentage points).

Given our results, mBHB traits presented sufficient genetic variance to be considered into the Spanish Holstein breeding program to select animals as an indicator of subclinical ketosis. To optimize computational efficiency, it may be sufficient to model mBHB in the first 3 lactations as separate traits and to perform genetic evaluations preferably using 2 test-day records collected within early lactation. This approach could lead to substantial cost savings without compromising genetic accuracy. For routine genetic evaluations, herd-year should be treated as a fixed effect provided a minimum threshold of records per group is ensured for all traits. Further research should explore the genetic relationships between mBHB, clinical ketosis, and other metabolic diseases, as well as the economic impact of metabolic health at the herd level.
